# Aging, Inflammation, and Comorbidity in Cancers—A General In Silico Study Exemplified by Myeloproliferative Malignancies

**DOI:** 10.3390/cancers15194806

**Published:** 2023-09-29

**Authors:** Johnny T. Ottesen, Morten Andersen

**Affiliations:** Mathematical Modeling—Human Health and Disease, IMFUFA, Department of Science and Environment, Roskilde University, 4000 Roskilde, Denmark; moan@ruc.dk

**Keywords:** cancer, immuno-competition, myeloproliferative neoplasms, aging, inflammation, comorbidity

## Abstract

**Simple Summary:**

Cancer escape links to the insufficient immune response. Thus, transition from a pre-cancerous state to cancerous development induced by morbidities competing for the immune resources is possible. We propose a simple but general conceptual model which suggests a unified explanation of cancer escape and other clinical and epidemiological observations, such as age-dependent prevalence and increased risk of getting cancer for patients with chronic inflammatory diseases. Myeloproliferative malignancy is used as a case throughout when specific data is considered but we conjecture the finding are general since they originate from immune deficiencies.

**Abstract:**

(1) Background: We consider dormant, pre-cancerous states prevented from developing into cancer by the immune system. Inflammatory morbidity may compromise the immune system and cause the pre-cancer to escape into an actual cancerous development. The immune deficiency described is general, but the results may vary across specific cancers due to different variances (2) Methods: We formulate a general conceptual model to perform rigorous in silico consequence analysis. Relevant existing data for myeloproliferative malignancies from the literature are used to calibrate the in silico computations. (3) Results and conclusions: The hypothesis suggests a common physiological origin for many clinical and epidemiological observations in relation to cancers in general. Examples are the observed age-dependent prevalence for hematopoietic cancers, a general mechanism-based explanation for why the risk of cancer increases with age, and how somatic mutations in general, and specifically seen in screenings of citizens, sometimes are non-increased or even decrease when followed over time. The conceptual model is used to characterize different groups of citizens and patients, describing different treatment responses and development scenarios.

## 1. Introduction

Pathogens—including somatic mutations—may be detected by antigen-presenting cells (APCs), which then display antigens on their surfaces, the major histocompatibility complex (MHC). The MHCs of the APCs are exposed to the naïve cells of the adaptive immune system, which consequently becomes activated. Such pathogen-specific effector cells multiply and migrate to the location of the pathogen, where they recognize and bind to the MHCs of the pathogens to initiate the elimination of these pathogens. The immune response may eradicate the pathogen to prevent uncontrolled growth. Uncontrolled growth demands medical intervention. However, in some cases, the immune response can keep the population of pathogens at an unseen level without eradicating the pathogen yet preventing uncontrolled growth for years. It has been speculated that such states may be in a temporary equilibrium, i.e., a dormant pre-cancerous state. In blood cancers, Clonal Hematopoiesis of Indeterminate Potential (CHIP) constitutes an example [1]. An allele burden above 2% of somatic mutations characteristic for hematopoietic cancers is observed in non-diseased citizens, with prevalence increasing from 1% to 10% with age [2,3]. In addition, a higher prevalence is found using more sensitive methods [4]. These citizens are characterized as having CHIP. Great variability is expected across different pathologies, due to the wealth of different pathogens (viruses, bacteria, fungus, malignant cells, sterile infection, etc.) However, the body is continuously exposed to pathogens and most threats are rejected or the pathogen is eradicated.

Single species such as malignant clones are mathematically well described by logistic or Gompertzian growth toward a carrying capacity [5]. The validity of such models is documented to fit various in vitro data as well as in vivo data well [6]. Whenever more than a single species is considered, the Lotka-Volterra competition model is frequently used [6]. It includes competition through the microenvironment, but solely in an implicit non-mechanistic way. Clone-to-clone competition may be mediated by, e.g., cytokines, chemokines, and effects of the innate immune system. Some of these mediators may be added to the models as additional terms [6,7]. The cost of such an extension is the increased complexity of the models and, consequently, a significant number of additional parameters, which mostly have unknown values. In computational medicine, informative mathematical models have a rich history [8], while in recent years the use of uninformative models known for example from AI, have attracted attention. Where the informative mathematical models deliver information about the underlying physiological mechanisms, the uninformed models do only deliver correlational results. On the other hand, uninformative models are superior in treating huge data sets. In this paper, we take an informative approach.

Recently, our focus has been the *JAK2V617F*-positive myeloproliferative neoplasms (MPNs), which are Philadelphia-negative hematopoietic cancer [9,10,11,12,13,14,15,16]. However, two important observations have not been fully understood quantitatively:

1. **the dormant CHIP observation.** In large screening studies of citizens, a significant fraction of the population carries the *JAK2V617F* mutation [17,18]. Re-examining these citizens 3–7 years later shows that the allele burden did not increase in approximately one-third of those who have not received an MPN diagnosis, while it had increased in two-thirds of these. [18] These citizens are asymptomatic regarding classical diagnostic criteria such as elevated blood cell counts, which is a severe risk of blood clots. Hence, such citizens are assumed to be in a pre-cancerous state, i.e., having CHIP. [12,18] Moreover, 38% of these are in a stable equilibrium CHIP state,
2. **the observation of linear increasing prevalence with age.** The prevalence of *JAK2V617F* MPN increases approximately linearly with age [19]. However, a quantitative explanation of why is lacking. More generally, it is known that inflammatory mediators are a key feature of aging and smoldering inflammation increases the risk of cancer progression [19,20].

CHIP is known to be associated with increased morbidity, such as coronary heart disease [21], chronic liver disease [22], chronic kidney disease [12,23], hematologic cancers [3,24], and potentially ulcerative colitis [25] and solid tumors [26]. From epidemiological studies, it is known that CHIP is overrepresented in cohorts with chronic inflammation, smoking [27,28,29], obesity [30,31], systemic lupus erythematosus [32], inflammatory cardiovascular diseases [33], and systemic sclerosis [34]. This supports the hypothesis of a strong relationship between CHIP and chronic inflammation. For more experimental evidence relating CHIP and inflammation see [2]. Evidence from animal models also supports the hypothesis that chronic inflammation impairs hematopoietic stem cell function, especially stem cell self-renewal [35,36,37,38,39,40]. This suggests the hypothesis that CHIP may be a precursor to hematopoietic cancer and couples to inflammation, which may, therefore, be a condition responsible for a transition from the pre-cancerous CHIP state to a cancerous development.

In the current work, we strive to answer both these questions by immuno-competition between concurrent immune-provocative diseases, one of these being hematopoietic cancer. These may be considered as comorbidities, where one may fertilize the growth of the other. The extra stress on the immune system from two different sources are in line with the decreasing potential hypothesis [41]. In some cases, the coupling may inhibit the growth corresponding to an immuno-stimulating interaction.

To study this, we will adopt a simple, but mechanisms-based approach to describe the cancer-infection-immune system mathematically. Specifically, we consider the *JAK2V617F*-positive MPNs and a general severe or sustained infection. The infection may be associated with a chronic auto-immune disease like Morbus-Crohn or with a COVID-19 virus infection. In all cases, the immune response mediates the coupling between the diseases, in this case, cancer and severe inflammation. To emphasize, our focus is cancer-related immuno-competition where comorbidities are each generating an inflammatory environment. All specific in silico investigations will use default parameter values for *JAK2V617F*-positive MPNs and COVID-19, but otherwise the considerations are general. See Appendix A for parameter values.

## 2. Materials and Methods

We use mathematical models to structure our basic clinical knowledge and hypothesis and to calculate the consequences of this knowledge and hypothesis using in silico tools. We will use the term virtual patients (VPs) for in silico models of subjects and patients in response to hypothetical perturbations, such as treatment or aging.

### 2.1. The Roskilde Immuno-Competition (RIC) Model

**Figure 1:**
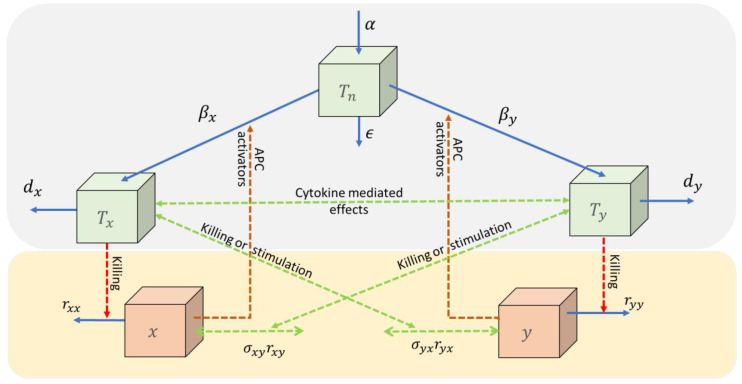
Two pathogens (x and y) activate the naïve T-cells ($T_{n}$ via antigen-presenting cells (APC) into specific effector cells ($T_{x}$ and $T_{y}$, respectively) which try to defeat the pathogens. All cell types may be eliminated with natural death rates and the pathogen by additional rates dependent on the number of specific effector cells. The naïve T-cells are upregulated by a baseline production (α). The pool of specific effector T-cells may affect each other through related cytokines, and these may expose an additional effect on the pathogens (green dotted curves). Such effects may be stimulating, neutral, or inhibiting. The mutual inhibiting case considered in this paper is denoted, the immune-competition model.

In [15], the immune-competition coupling is through a limitation on the size of the naïve T-cell per day. The adaptive immune response is activated by pathogens and transformed cells; thus, pathogen-specific effector T-cells (CD8+) become activated to fight the pathogen. The RIC model includes naïve T-cells, regulatory cytokines, and cytotoxic effector T-cells, see Figure 1.

Assuming the fast parts of the dynamics take place instantaneously (mathematically, a quasi-steady state approximation), the coupled disease progression may be described by two coupled ordinary differential equations,

(1) $$x^{\prime }=a_{x}x\left(1-\frac{x}{K_{x}}\right)-\frac{\alpha r_{x}p_{x}\beta_{x}}{d_{x}\varepsilon }\frac{x^{2}}{\left(\frac{\beta_{x}}{\varepsilon }x+\frac{\beta_{y}}{\varepsilon }y+1\right)\left(\frac{r_{x}\left(1-p_{x}\right)}{d_{x}}x+1\right)},$$

and

(2) $$y^{\prime }=a_{y}y\left(1-\frac{y}{K_{y}}\right)-\frac{\alpha r_{y}p_{Y}\beta_{y}}{d_{y}\varepsilon }\frac{y^{2}}{\left(\frac{\beta_{x}}{\varepsilon }x+\frac{\beta_{y}}{\varepsilon }y+1\right)\left(\frac{r_{y}\left(1-p_{y}\right)}{d_{y}}y+1\right)}.$$

Here, $x^{\prime }$ and $y^{\prime }$ denote the rate of change of the respective diseases of size x and y, $a_{x}$ and $a_{y}$ the intrinsic growth rates, $K_{x}$ and $K_{y}$ the intrinsic carrying capacities, α and ε the baseline production rate and elimination rate of naïve T-cells, $\beta_{x}$ and $\beta_{y}$ the per capita production rates of disease-specific effector cells, and $p_{x}$ and $p_{y}$ denote the probability of apoptosis caused by the respective effector cells.

### 2.2. Dimensionless RIC Model

By normalizing the variables $X=\frac{x}{K_{x}}$ and $Y=\frac{y}{K_{y}}$ and clustering the parameters into a reduced number of new parameters:

$$A_{1}=\frac{\alpha r_{x}p_{x}}{a_{x}d_{x}}A_{2},A_{2}=\frac{\beta_{x}K_{x}}{\varepsilon },A_{3}=\frac{\beta_{y}K_{y}}{\varepsilon },A_{4}=\frac{r_{x}K_{x}\left(1-p_{x}\right)}{d_{x}},\;B_{0}=\frac{a_{y}}{a_{x}},B_{1}=\frac{\alpha r_{y}p_{y}}{a_{x}d_{y}}A_{3},B_{4}=\frac{r_{y}K_{y}\left(1-p_{y}\right)}{d_{y}},$$

the RIC model reduces to,

(3) $$X^{\prime }=X\left(1-X\right)-A_{1}\frac{X^{2}}{\left(A_{2}X+A_{3}Y+1\right)\left(A_{4}X+1\right)},$$

and

(4) $$Y^{\prime }=B_{0}Y\left(1-Y\right)-B_{1}\frac{Y^{2}}{\left(A_{2}X+A_{3}Y+1\right)\left(B_{4}Y+1\right)},$$

where prime denotes the derivative with respect to the dimensionless time $T=a_{x}t$.

The RIC model guarantees that solutions cannot become negative if they initially start with non-negative values and if for some reason X>1 or Y>1, $\left(X,Y\right)$ will return to the compact unit square $\left[0,1\right]\times \left[0,1\right]$ in a finite time. Moreover, if $\left(X,Y\right)$ is inside the compact unit square, it cannot leave it again. Thus, the unit square constitutes an attracting trapping region, and all interesting dynamics happen inside this box.

Remark, a pathogen is eradicated if and only if the right-hand side of the respective differential equation becomes negative. For x=1, this happens if $a_{x}\left(1-\frac{1}{K_{x}}\right)<\frac{\alpha r_{x}p_{x}\beta_{x}}{d_{x}\varepsilon }\frac{1}{\left(\frac{\beta_{x}}{\varepsilon }+1\right)\left(\frac{r_{x}\left(1-p_{x}\right)}{d_{x}}+1\right)}$.

Methods from the mathematical discipline of Dynamical Systems are used to analyze the mathematical model. These methods include phase plane analysis, steady states, stability, separatrix, and basin of attraction, etc. [5,6,7]. The results are general across cancers, except when specific parameter values are used, which relate to specific malignancies; denoted as qualitative analysis versus quantitative analysis in mathematics [5,7]. As posed by the model, all variables describing disease load will be in dimensionless/normalized units unless otherwise specified.

### 2.3. Data

From the Copenhagen General Population Study, data were collected from screening of 49,488 citizens in Copenhagen, Denmark, obtained in the period 2003–2008 [18]. Of these, 63 were *JAK2V617* mutation-positive and the surviving 52 were re-invited for a follow-up examination in 2012, and 26 of these were undiagnosed. Out of these, 18 became MPN diagnosed at the re-examination, while the remaining 8 did show symptoms and were left undiagnosed [18]. From the GESUS screening of 29,958 citizens in the Zealand region of Denmark [17,19], prevalence data on having acquired a malignant *JAK2V617F* mutation is reported in [19].

## 3. Results and Discussion

Interpreting the findings through the RIC model is considered a part of the results.

### 3.1. The RIC Model

The conceptual model consists of naïve T-cells, two pathogens, and corresponding specific effector T-cells as variables (quantities that change over time) and fourteen physiological parameters (quantities that do not change or vary slowly over time), see Figure 1. One may reduce the model to a dimensionless form and perform a quasi-steady state approximation whereby it reduces to two variables, in this case the pathogens, and seven clusters of parameters. For the derivation of the equation, see Section 2.

Generally, the cross interactions from one immune response to the growth of the other disease may be inhibiting, neutral, or stimulating. In principle, this gives nine possible cases. However, in the present work, we will focus solely on the case of mutual competition, i.e., the immune response provoked by each disease inhibits the other.

Below we focus on phenotypes and use the RIC model to analyze the progression of comorbidities, discuss possible treatments, and investigate the effect of aging. The phenotypes considered have agonistic immuno-effects, and such VPs ultimately develop comorbidities.

Seven clusters of parameters govern the coupled progression of the RIC model and thus contain important information about the system dynamics. These clustered parameters consist of fourteen physiological parameters. $A_{4}$ and $B_{4}$ are the ratios of the strengths of the second-order elimination of specific effector T-cells to their natural death rates. $A_{2}$ and $A_{3}$ denote the ratio of the per capita rate of production of the respective effector T-cells to the natural death rates of the naïve T-cells. $A_{1}$ and $B_{1}$ are the ratios of the baseline production of the naïve T-cells to the growth rate of the pathogens multiplied by the ratio of the binding reaction rate multiplied by the probability of the pathogen being eliminated to the natural death rate of the specific effector T-cells (times $A_{2}$ and $A_{3}$, respectively). $B_{0}$ is the ratio between the intrinsic growth rates of the infection and that of cancer (see Section 2 for further details).

The dynamics of each disease are described by a normalized logistic growth term and a term describing the effect of the effector T-cells. The strength of the immune response is given by $A_{1}$ and $B_{1}$, respectively. These responses are inhibited by a limited capacity of the naïve T-cells and elimination of the pathogen-specific effector T-cells (see Section 2 for further details).

### 3.2. In Silico Dynamics of Single Disease Progression

**Figure 2:**
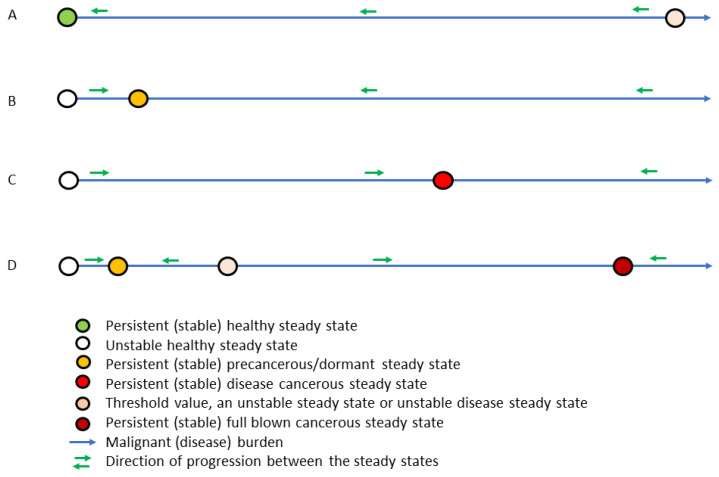
For a single disease, in this case cancer, four persistent (stable) steady states exist toward which all other states move over time indicated by green arrows. In between these persistent (stable) steady states, threshold states exist. Depending on the disease and the immune parameter values four cases exist, the healthy case (**A**), the precancerous dormant case (**B**), the mild cancerous case (**C**), and the co-existing dormant fatal cancerous case (**D**). In the last case, the state ultimately approaches the dormant state or the fatal cancerous state. After having a single mutation, in this case, the immune system controls the clone and the state develops into the dormant state but after a severe perturbation crossing the threshold value, the state develops into fatal cancer.

For simplicity, we start by considering an idealized universe where only one disease exists, say cancer assuming a single mutation has taken place. If the immune system is strong enough the cancer will be eradicated and the outbreak will never be realized (Figure 2A). This happens if the right-hand side of Equation (1) is negative. For x=1 and $y=K_{y}$, it happens if

(5) $$a_{x}\left(1-\frac{1}{K_{x}}\right)<\frac{\alpha r_{x}p_{x}\beta_{x}}{d_{x}\varepsilon }\frac{1}{\left(\frac{\beta_{x}}{\varepsilon }+1\right)\left(\frac{r_{x}\left(1-p_{x}\right)}{d_{x}}+1\right)}.$$

We denote an immune system with such a constellation of parameter values associated with the specific disease as a safe immune response. Thus, having a safe immune response makes the subject immune to the associated disease since it will be eradicated as soon as it is initiated. A safe immune system may require extreme parameter values, dependent on the specific disease.

If the immune system is not safe, then any perturbation, whatever small, will result in a non-decreasing amount of malignant cells (Figure 2B–D). That is to say, the healthy steady state is unstable (an unstable saddle point, to be mathematically precise) and ultimately all neighboring states in the unit square are repelled away from it. Consequently, such perturbation—if not too big—will force the state to approach a persistent steady state (mathematically, this is denoted as a stable steady state) being either a precancerous state (Figure 2B,D) or a mild disease cancerous state (Figure 2C,D). From here on, two possibilities arise, either any perturbation of such state ultimately returns toward the state, meaning that no other persistent (stable) steady state exists (Figure 2B,C), or a large perturbation may let the state grow further (Figure 2D). In that case, the perturbation needs to let the state cross a threshold value, an unstable steady state, and the state will be attracted toward a persistent (stable) fatal disease steady state. Thus, the former state with fewer malignant cells will be denoted as a dormant state. In this case, the space of all states is divided into two connected regions, one region where all states are attracted toward the persistent (stable) dormant state and another region where all states are attracted toward the persistent (stable) fatal disease state. Hence the system cannot without a substantial external perturbation, reach the persistent (stable) fatal disease state. See Figure 2.

### 3.3. In Silico Dynamics of Comorbidity Progression

We now drop the unrealistic presumption of an ideal universe with only one disease and consider the situation of potential cancer and severe infection. The right way to understand the dynamical development of the system of diseases in silico is to used the phase space (a phase line in case of a single disease and a phase plane in case of comorbidities). A phase space shows the disease load on the axis but does not specifically have a time axis. We elaborate on this below.

Assuming an inefficient immune response, i.e., a non-safe immune response, the healthy steady state of no disease becomes unstable, meaning that any perturbation will initiate the disease progression but possibly toward an unseen burden. In the absence of comorbidity, a disease may appear with either low (Figure 2B,D) or high (Figure 2C,D) disease burden. In case where both states co-exist while still disallowing comorbidity, the two persistent (stable) steady states exist, separated by a threshold corresponding to an unstable steady state (Figure 2D). Thus, the disease initiation naturally progresses toward the low disease level. However, allowing comorbidities, all these mono-disease steady states are unstable, meaning that any small perturbations with the comorbidity will cause the initiated disease to depart away from these steady states. Thus, comorbidity whenever initiated, is the common situation. The diseases may be fully eradicated only in the case of a very strong immune response.

**Figure 3:**
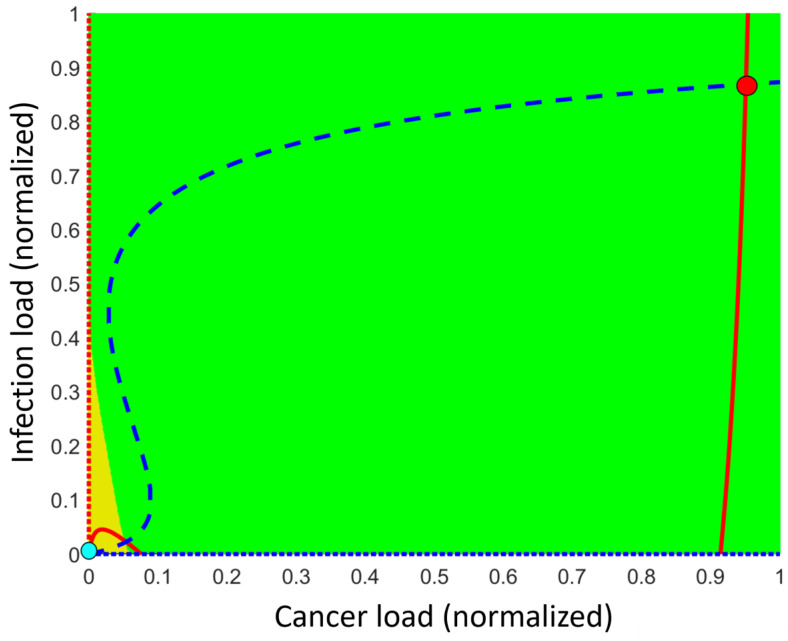
Typical phase plane having cancer load on the first axis and infectious load on the second axis using the default parameter values listed in Appendix A. The normalized disease burdens are on each axis and a given point in the plane describes a coupled disease state. Two persistent (stable) comorbidity steady states may exist, a high disease burden state (red dot at the upper right) and a low disease burden state (cyan dot at lower left). The (full and dotted) red curves are the nullclines for disease x (where $x^{\prime }=0$) while the blue (dotted and dotted) curves are the nullcline for disease y (where $y^{\prime }=0$). Steady states are where these nullclines intersect. The third intersection away from the axis is an unstable comorbidity steady state lying on the threshold curve (separatrix), which separates the basin of attraction related to the low disease burden comorbidity state (triangle colored orange) from the basin of attraction related to the high disease burden comorbidity state (colored green). Additional unstable non-comorbidity steady states may exist on the axis.

The best way to understand the progression of the comorbidity system consisting of potential cancers and severe infections is to introduce the phase space. A phase space is simply an illustration showing the diseases and how these change over time, but without showing a time axis specifically. Figure 2 is a simple one-dimensional phase space. Allowing two diseases comes with a price, since it requires a two-dimensional phase space, which has the cancer burden on one axis and the infection burden on the other axis, but no specific time axis; see Figure 3. A stationary comorbidity state of a subject would then be a point in this space. If the state changes over time, it spans a trajectory in this phase space. However, time will not appear explicitly. It is common to mark the persistent (stable) steady states and color the region around these wherein all states approach the persistent (stable) steady state over time. Such regions are denoted as basins of attractions associated with the persistent (stable) steady state in the region. The basins of attractions divide the plane into non-overlapping connected regions (Figure 3). We emphasize that mono-disease states lie on the axis, the healthy state is where these intersect, and the comorbidity states lie in the plane away from the axis (Figure 3).

In the following, we will focus solely on the persistent (stable) comorbidity states. The exact number of persistent (stable) comorbidity steady states depends on the specific values of the parameters, but there are always one or two such states. In the case of one persistent (stable) comorbidity steady state, all other states in this region away from the axis move toward it over time. This may be a persistent (stable) dormant steady state or a persistent (stable) fatal disease steady state, depending on the parameter values. In the case where two persistent comorbidity steady states exist, an unstable steady state lies in between these. More precisely, the unstable steady state lies on a threshold curve (mathematically denoted as a separatrix) dividing the (phase) plane into two parts, the basin of attractions (see Figure 3). In the lower-left basin of attraction, the persistent (stable) low disease burden comorbidity steady state lies and all other states in this basin of attraction are attracted toward it over time. The same holds for the other basin of attraction, where the persistent (stable) high disease burden comorbidity steady state lies. All other states in this basin are attracted toward this persistent steady state over time. Figure 3 illustrates the case of two persistent (stable) steady states.

For the default parameter values (see Appendix A), two persistent (stable) comorbidity steady states exist. Hence, the system exhibits bistability. One of these persistent (stable) states is generally associated with low disease burden while the other is generally associated with high disease burden, corresponding to an undiagnosed dormant state and a full-disease state, respectively (see Figure 3). The exact position of the persistent (stable) comorbidity steady state and the threshold curve (separatrix) separating the basins of attractions depend on the specific values of the parameters. By varying these parameter values, the situation mostly changes smoothly, but at a few specific values, it changes abruptly, which mathematically corresponds to bifurcations. The value of the varying parameter where such an abrupt change happens is denoted as a bifurcation point with respect to that parameter. At such bifurcation points, one of the two persistent states merges with the threshold curve (separatrix) and these are annihilated, thus only the other persistent (stable) steady state remains and over time this persistent (stable) steady state attracts all other comorbidity states lying away from the axis. Two different bifurcation points exist, one where the dormant steady state merges with the threshold curve (separatrix) and another one where it is the fatal disease state that merges with the threshold curve (separatrix). Thus, varying parameter values may switch from bi-stability to mono-stability and vice versa. As an illustrative example, let the baseline production α of naïve T-cells be the varying parameter. A bifurcation takes place for large values leaving behind the persistent (stable) dormant steady state as the only remaining persistent (stable) steady state, while another bifurcation happens for small values, leaving behind the persistent (stable) fatal state as the only persistent (stable) steady state. We expand on this below.

### 3.4. Data Comparison with the RIC Model

**Figure 4:**
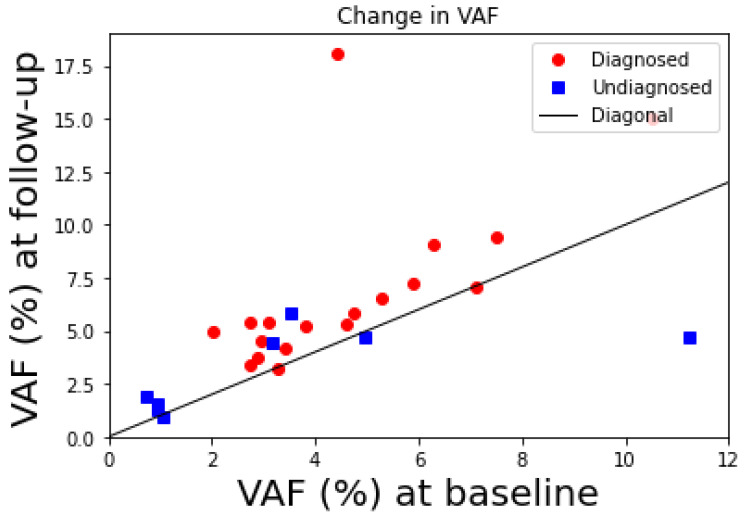
Increase in *JAK2V617F* variant allele burden (VAF in percentage) from baseline measurement (x-axis) to follow-up (y-axis) measurement. Red dots are those acquiring the diagnosis at the follow-up re-examination and blue squares are those who did not. Thus, 3 out of 8 undiagnosed citizens lie under the diagonal (black line) representing points where the follow-up values would equal the baseline values. Thus, these 3 had a decrease in allele burden from baseline to follow-up corresponding to 38% of the citizens who have not acquired an MPN diagnosis. Data from [14].

In hematological malignancies, the persistent (stable) dormant steady state, likely being undiagnosed, may correspond to clonal hematopoiesis of indeterminate potential (CHIP), a dormant pre-cancerous state. From screenings of large cohorts of citizens, temporary persistent dormant steady states have frequently been observed [18]. See Figure 4.

In the Copenhagen General Population Study, 49,488 citizens in Copenhagen, Denmark, were screened for the *JAK2V617F* mutation during 2003–2008, and 63 were *JAK2V617* mutation positive (>1% *JAK2V617* variant allele burden) [18]. In 2012, 52 of these were re-invited for a follow-up examination (11 deaths before re-examination) and 26 of these were undiagnosed at the time of the re-examination, but 18 of these became subsequently diagnosed with an MPN diagnosis (15 ET, 2PV, 1PMF), while the remaining 8 did show symptoms and were left undiagnosed. Out of these 5 showed a modest increase in allele burden while 3 showed a decrease from baseline to follow-up.

We interpret these examinations as up to 38% of the CHIP citizens were in a persistent (stable) dormant steady state. These findings are illustrated in Figure 4 showing the baseline values and the follow-up values for the *JAK2V617F* allele burden of the undiagnosed twenty-six CHIP citizens who had a follow-up examination. Thus, around 38% of the CHIP citizens did not have a significant increase in the *JAK2V617F* allele burden and were therefore considered to be in a persistent (stable) dormant steady state. It is also remarkable that two of the eighteen citizens diagnosed due to symptoms at the follow-up had a modest decline in *JAK2V617F* allele burden too.

### 3.5. Classification of VP Subtypes

We may group the VPs into four groups, those with a very strong immune response (denoted healthy- or H-subtype), those where a single but dormant comorbidity state exists (dormant- or D-subtype), those where a single but fatal comorbidity state exists (fatal- or F-subtype), and those where two comorbidity disease burden exist (risk- or R-subtype). Whether a VP belongs to which subtype depends on the parameter values for the VP. We emphasize that these subtypes are also disease-dependent, meaning that for any two specific comorbidities, a VP may belong to one of the subtypes but for other comorbidities, the same VP may belong to another subtype. Thus, the subtypes depend on both VP-specific parameters as well as on the diseases considered.

The H-subtype VPs are quite safe regarding not only comorbidity progression but also disease progression.

Imagine a VP of R-subtype who had an initial mutation and a minor infection, and then the disease burden grows. Such comorbidity develops logistically over time and approaches the persistent (stable) comorbidity steady state, likely of low disease burden. Afterwards, the system will stay in this dormant state if not severely perturbed. A dormant state may be very low in disease burden and therefore hardly realized and therefore often be undiagnosed. We interpret this as a pre-cancerous state accompanied by a little inflammation. In case the disease burden is not very low, the disease may be realized and diagnosed as a dormant disease state, e.g., cancer that temporarily does not progress further. A severe perturbation may let the comorbidity cross the threshold curve (separatrix), and if left untreated, a fatal comorbidity develops, i.e., the disease burden for the VP will progress toward a high disease burden, which ultimately will be diagnosed and treated.

As mentioned, the exact location of these persistent (stable) comorbidity steady states and the threshold curve (separatrix) depend on the specific parameter values. Changing the parameter values slowly or stepwise may move the location of the threshold curve (separatrix) and the persistent (stable) comorbidity steady states. The location of the threshold curve (separatrix) may move outside the region of interest (constituted of the comorbidity square), where each disease is between zero disease burden and the maximal disease burden (the carrying capacity). Hence, the system is transformed into a D- or an F-subtype dependent on the parameter values; i.e., a VP with exactly one persistent (stable) comorbidity steady state.

For the D- and F-subtypes, all comorbidity states will approach the unique but parameter-dependent persistent (stable) comorbidity steady state over time. The location of such a persistent (stable) comorbidity steady state may be anywhere in the unit square.

### 3.6. The Three E’s of Immunoediting

The model explains the three E’s of immunoediting, elimination, equilibrium, and escape. For the F-subtype, the equilibrium phase does not exist, and the disease goes directly into the escape phase. For the D-subtype, the persistent comorbidity steady state is likely a low disease burden state. For the R-subtype, a transition from equilibrium to escape needs a perturbation in disease burden such that it crosses the threshold curve (separatrix).

Severe infection by e.g., COVID-19 may be a way to perturb the system and obtain a transition from H- to D-subtype, from H- to R-subtype, from D- to R-subtype, or from D- to F-subtype, see [15]. Another mechanism is gliding in the parameter values, like the age-dependent baseline production rate α of naïve T-cells, which may lead to a bifurcation creating a similar transition (see more below). Finally, exhaustion of the specific effector T-cells may lead to escape. A cascade of cytokines, chemokines, and leukocytes is activated when the adaptive immune system is engaged, as seen for the *JAK2V617F* mutated cells MPNs [42]. If such inflammation is not controlled, it may affect the system parameters and lead to the progression of chronic inflammatory diseases. Consequently, escape may result.

As the low disease burden comorbidity state may escape to fatal disease, the possibility of reversing the transition by treatment may appear favorable. For the R-subtype, one may lower the disease burden by moving the persistent (stable) high disease comorbidity state appropriately or infer a transition to a D-subtype. If the disease state can be treated down from the basin of attraction associated with the persistent (stable) fatal comorbidity steady state of high disease burden to the basin of attraction for the persistent (stable) dormant comorbidity steady state of low disease burden, by crossing the threshold curve (separatrix) in the favorable direction, the natural immune response will subsequently secure the state to approach the persistent (stable) dormant comorbidity steady state with low disease burden over time. However, this needs to be done in due time to ensure that the treatment can force the trajectory to cross the separatrix [15].

### 3.7. Aging Causes Immuno-Deficiency in Cancer Development 

**Figure 5:**
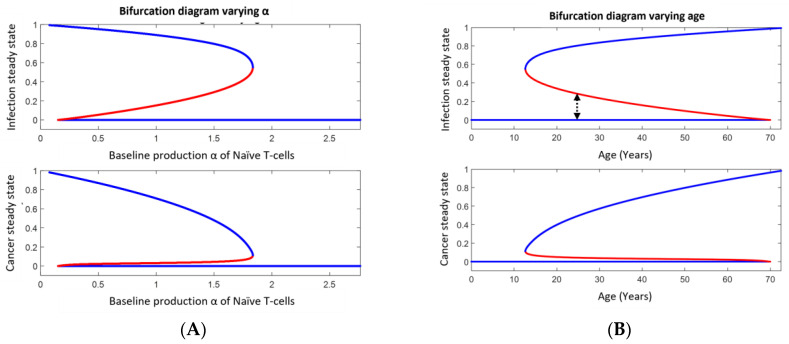

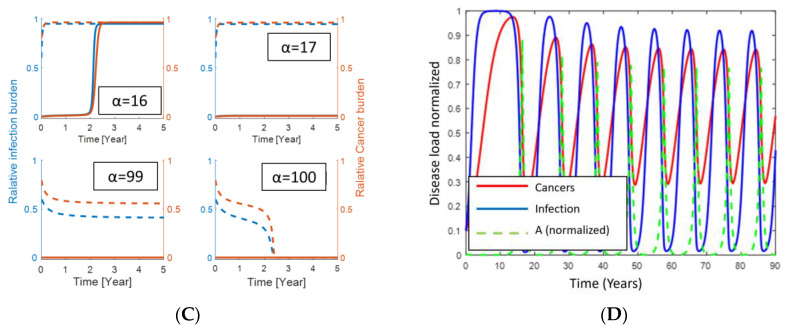
Upper panel in (**A**) shows the bifurcation diagram for how the persistent (stable) dormant infection (lower blue curve), the persistent (stable) fatal infection (upper blue curve), and the infection burden at the threshold curve (separatrix, middle red curve) varies with the baseline production (α) of naïve T-cells. The lower panel shows the similarity for cancer instead of infection. Panel (**B**) shows a similar diagram as in panel (**A**) but instead, as a function of age assuming α decreases linearly with age. The distance between the dormant infection curve and the threshold curve (indicated by the black dotted double arrow at year 25 as an example) is inversely proportional to the probability of disease escape due to infection. Panel (**C**) illustrates how cancer and infection progress over time for different perturbation corresponding to the initial value at time 0. The dotted curves start with a high disease burden (near the persistent fatal disease steady state) while the full curves start with a low disease burden (near the persistent (stable) steady state). The red curves and axis show the relative cancer burden while the blue curves and axis show the infection burden. From upper left to lower right, α = 16, 17, 99, 100. Panel (**D**) shows a hypothetical treatment scenario for an elderly VP with disease progression toward fatal disease (low value of α). First, disease escape is seen (no treatment), thus treatment is applied by increasing α (shown as the green dotted curve). This results in a decrease in disease burden. When the VP is in the basin of attraction for the persistent (stable) dormant state, the VP is ‘cured’. However, stopping the treatment will likely let α approach its low value again and history repeats.

Aging affects the inflammatory response like persistent infections [43]. Evidence shows the production of naïve T-cells declines with age [43], which in the RIC model is represented by a decline in α. If the VP is not of H-subtype, comorbidity may emerge. From the phase space in Figure 3, we may consider the vertical distance (that is, inflammation on the second axis) and the horizontal distance (that is, cancer on the first axis) between the steady states. These are shown in Figure 5A as a function of the baseline production rate α of naïve T-cells. Figure 5B shows essentially the same but as a function of age. Typical examples where diseases are plotted versus time are depicted in Figure 5C while a potential treatment scenario over time is shown in Figure 5D. In more details and using the default parameter values in Appendix A, the situation is as follows (see Figure 5); or extreme parameter values fulfilling inequality (5) only the persistent (stable) healthy steady state exists. Otherwise, for large values of α, i.e., α > 100, only the dormant steady state exists among the persistent (stable) comorbidity steady states. This corresponds to a D-subtype and is expectably typical at young ages. Being the only persistent (stable) steady state in the region of interest (i.e., in the open unit square for the dimensionless variables), all co-existing states are attracted toward this state since no limit cycle can exist. For 17 < α < 99, both the persistent (stable) dormant state and persistent (stable) fatal disease comorbidity steady state exist, separated by the threshold curve (separatrix) separating the basins of attractions. All comorbidity states below the separatrix are attracted toward the persistent (stable) dormant steady state, while those states above the threshold curve (separatrix) are attracted toward the persistent (stable) fatal disease steady state as time evolves. As a function of α, the unstable steady state at the separatrix approached the persistent (stable) dormant states as α decreases, and the two merge approximately for α = 16 and consequently annihilate. Mathematically this is a saddle-node bifurcation [5,7]. The unstable steady state on the separatrix merges with the persistent (stable) fatal disease steady state when α increases to approximately 99 in another saddle-node bifurcation. For 17 < α < 99, the persistent (stable) fatal comorbidity steady state co-existing with the persistent (stable) dormant comorbidity steady state. For α < 16, the persistent (stable) fatal disease comorbidity steady state is the only existing persistent (stable) comorbidity steady state. Thus, for α < 16 all states are attracted toward the persistent (stable) fatal disease comorbidity steady state. The last situation corresponds likely to elderly people. The bi-stable situation corresponds mostly to people between young and elderly. This is all summarized in Figure 5A,B and examples of representative solutions showing the disease burdens over time appear in Figure 5C.

### 3.8. Infection May Cause Immuno-Deficiency and Consequently Cancer Escape

Notice, the threshold curve (separatrix) mostly lies much nearer to the persistent (stable) dormant comorbidity steady states than to the persistent (stable) fatal disease comorbidity steady states. This means that a relatively little perturbation may force the states in the basin of attraction for the persistent (stable) dormant state to exceed the threshold curve (separatrix). Hence, the system jumps from the basin of attraction for the persistent (stable) dormant disease comorbidity steady state to the basin of attraction for the persistent (stable) fatal disease comorbidity steady state and thereby initiates a disease escape. Moreover, the persistent (stable) dormant comorbidity steady state approaches the threshold curve (separatrix) as people become elderly, which means that even a smaller perturbation may cause disease escape. Thus, elderly people become more prone to disease escape triggered by, for example, infection.

### 3.9. Aging Explains the Observed Prevalence of JAK2V617F MPNs

We have analyzed the possibility of cancer escape by infection in Section 3.7. Considering a continuous infection load, the probability of escape will be inversely proportional to the distance from the persistent (stable) dormant state to the threshold curve (separatrix) along the axis of relative infection, see Figure 5A. This distance changes with α and thus with age as seen in Figure 5B [43].

**Figure 6:**
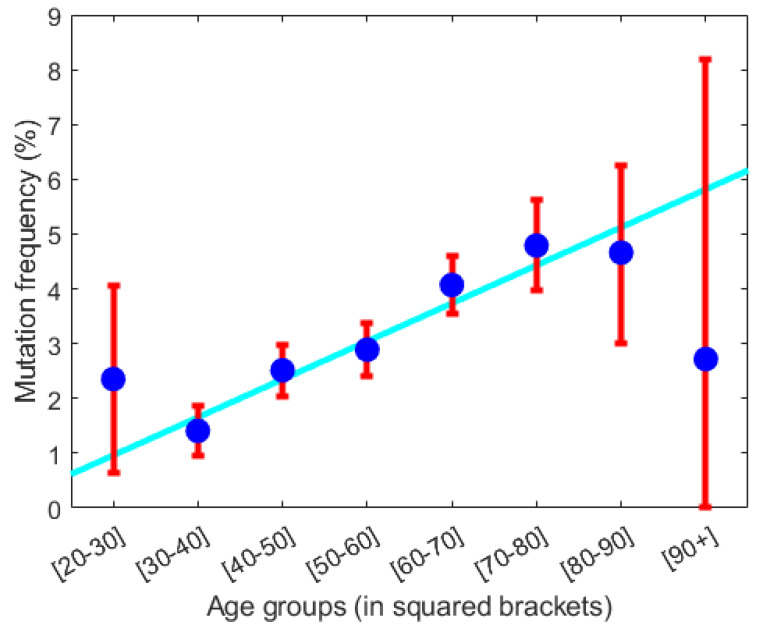
From the GESUS screening of 19,958 citizens in the Zealand region of Denmark the prevalence of having acquired a malignant *JAK2V617F* mutation is calculated. The prevalence in percentages is stratified into age decades (shown in squared bracket on the x-axis) as shown by blue dots with standard deviation given by the red whiskers. The cyan-colored line is the best linear fit to data neglecting the first and last data points. The best fit has a slope of approximately 0.1%. Thus, the prevalence increases approximately linearly with 0.1% per year or 1% per decade. Adopted from [19].

From the prevalence data in Figure 6, it follows that the annual increase in prevalence due to aging is approximately 0.1%. The inverse of the distance from the infection burden of the persisting (stable) dormant steady state to the infection burden of the threshold curve (see Figure 6) is proportional to the probability of disease escape caused by infection. This probability approximately increases linearly. From Figure 6, disease escape happens with a probability, which approximately increases by 0.2% per year due to infection. If we assume the increase in prevalence is due to disease escape caused by infection, the yearly increase in probability 0.2% should equal the yearly increase in prevalence which is approximately 0.1%. Considering the uncertainties in the calculations, this is not a significant difference.

### 3.10. Reversing Disease Progression in VPs by Naïve T-Cell Therapy

The question ‘What if we could manipulate α clinically?’ suggests itself, since it corresponds to making a subject younger immunologically. To make the scenario comprehensive we continue with the specific parameters from our default data. Consider a young subject while getting older. Ultimately disease escape will happen. Thus, a treatment increasing α to above 100 would cure the patient over time. However, pausing the treatment would cause a relapse since we expect α to reapproach its previous value below 16. However, the relapse would not appear until α falls below 16. In Figure 5D, we specifically assume α to decline exponentially with a negative rate of −4 toward 0.15 between the hypothetical treatments and to grow toward 100 as one minus a decaying exponential with a negative rate of −3 during the hypothetical treatments. Repeatedly treating the patient and pausing the treatment, after an on-off scheme according to the level of cancer, results in an oscillatory disease pattern as illustrated in Figure 5D. We emphasize that immuno-aging is individual, since the subject-specific parameters are so. Thus, a person with a lower age may have a higher immuno-age than another person of a higher age.

Consider a fatal diseased VP, with an α between 17 and 99, being treated by some conventional treatment and who responds well. Pausing treatment may result in a relapse, often earlier than desired. In Figure 5A, it is seen that the disease needs to be treated to a low disease burden if α is far away from 100. Thus, combining the treatment with a treatment targeting immuno-aging, may result in crossing the separatrix curve, and hence introducing a more complete treatment than mono-drug therapy. Of course, another perturbation may reintroduce disease progression and similarly aging ultimately will.

## 4. Conclusions

The informative physiological RIC model serves as a comprehensive tool to understand the progression of cancer phenotypes related to inflammation showing agonistic immuno-effects. Thus, the progression of cancer-inflammatory comorbidities can also be understood through the RIC model. Taken further, the understanding of such couplings may help in better understanding hurdles during treatments.

The RIC model comprehensively classifies VPs into four subtypes, the H-, D-, F-, and R-subtype. The subtypes are disease-specific and subject-specific since they depend on the subject-specific parameter values. These have different prognoses. Subjects of the H-subtype are safe since any initiation of the considered diseases will be eradicated immediately. The D-subtypes will approach a persistent (stable) dormant state over time, likely with a relatively low disease burden. The F-subtypes will approach a persistent (stable) fatal state over time, with a relatively high disease burden. The R-subtypes will first approach a persistent (stable) dormant state over time consisting of a low disease burden. However, a large perturbation may cause it to subsequently progress toward a persistent (stable) fatal state. Such perturbation may be caused by a severe infection or the origination of a chronic auto-immune disease. In some cases, for a subset of parameter values, an even smaller perturbation may lead to disease escape. Such perturbation may be smoking or seasonal flu.

Other important perturbations are those which cause a glide in the parameter values. Specifically, we studied the effect of changing the baseline production rate α of naïve T-cells. Assuming very large or extreme values of α, a subject will be of H-subtype. If the value of α decreases, the subtype eventually changes and transforms the VP into a D-subtype. Continuing the decrease in α, an abrupt change ultimately happens at a VP-specific value for α, transforming the VP into an R-subtype. Further decreasing α ultimately results in another abrupt change, transforming the VP into an F-subtype. We associate this development with immuno-aging. Mathematically, the abrupt changes are bifurcations.

The above immune-aging development for a VP reflects most likely the history of a young person becoming elderly. Aging is associated inversely with immuno-aging [20,43]. Thus, the above gliding in α corresponds, in general to aging. If diagnosed diseases are mostly due to R-subtypes, an additional inflammation may have caused the transition from the basin of attraction of the persistent (stable) dormant state to the basin of attraction of the persistent (stable) fatal steady state. The probability of such a transition is inversely proportional to the distance between the inflammation at the persistent (stable) dormant steady state and that of the threshold curve vertically above the state. Hereby, the probability of disease progression is estimated to be 0.2% per year, which is supported by the estimate of 0.1% from the prevalence study in [43]. All of this aligns with the updated ‘hallmarks of cancers’ from 2011 where ‘avoiding immune destruction’ became a dominant paradigm [44].

The persistent (stable) dormant steady states for the D- and R-subtypes suggest that some pre-cancerous subjects have a mutational burden in temporary equilibrium, which is observed in large population screening programs in Denmark. Independently *JAK2V617F* allele burden data from [18] confirm such temporary equilibrium in 15% of the screened CHIP citizens and 38% of the re-examined CHIP citizens.

Some mathematical models have been proposed to describe cancer-immune interactions. Most of these focus on specific treatments [45,46,47,48,49], while others are more general but come with the price of relatively high complexity, such as a large number of hardly determinable parameter values in practice [50]. All include several ad-hoc motivated empirical Michaelis-Menten factors, which are rarely based on underlying mechanisms. In contrast, we have proposed a very simple, mechanisms-based model with only a very limited number of clustered parameters consisting of products of physiological parameters. The strength of the RIC model is its ability to unify explanations for a variety of clinically observed phenomena. Thus, the conceptual model may add to increasing the understanding of cancer in general.

## Data Availability

See references.

## Appendix A

Tables show physiological parameter values from the literature with our choice of parameter values included. These vary significantly depending on the type of cancer and infection considered. The parameter values are person-specific as well as disease-specific.

**Table A1:** Physiological parameter values.

**Parameter**	$a_{x}$	$K_{x}$	$\beta_{x}$	$d_{x}$	$r_{x}p_{x}$
**Units**	**day^−1^**	**cell**	**cell^−1^ day^−1^**	**day^−1^**	**cell^−1^ day^−1^**
Jafari et al. [51]	1.05	455	33.3	0.12	0.015
Gil et al. [52]	0.01	10^12^	10^12^	-	5 × 10^−11^
Makhlouf et al. [53]	4.31 × 10^−1^	10^9^	1 × 10^7^	4.12 × 10^−2^	1.1 × 10^−7^
Unni and Seshaiyer [54]	4.31 × 10^−1^	5 × 10^7^	1 × 10^6^	4.12 × 10^−2^	3.5 × 10^−6^
Gurcan et al. [55]	0.18	5 × 10^6^	10	4.12 × 10^−2^	4.401 × 10^−8^
Khajanchi et al. [56]	0.18	1 × 10^6^	-	0.412	-
Pillis et al. [57]	5.14 × 10^−1^	10^9^	1 × 10^7^	4.12 × 10^−2^	3.50 × 10^−6^
Kuznetsov et al. [58]	0.18	5 × 10^8^	7.7 × 10^6^	-	-
Pillis et al. [59]	1.5	1	1	-	0.5
Kuznetsov et al. [60]	0.1877	5.319 × 10^2^	-	-	-
Sajid et al. [61]	1.15 × 10^−2^	10^5^	-	-	-
Ottesen et al. [62]	1.3 × 10^−3^	3 × 10^4^	-	-	-
Our default value	5 × 10^−2^	3 × 10^4^	0.125	1 × 10^−2^	2 × 10^−4^
**Parameters**	$r_{x}$	$p_{x}$	$r_{x}\left(1-p_{x}\right)$	α	ε
**Units**	**cell^−1^ day^−1^**	**-**	**cell^−1^ day^−1^**	**cell day^−1^**	**day^−1^**
Jafari et al. [51]	-	-	-	-	-
Gil et al. [52]	-	-	10^−13^	3 × 10^5^	10^3^
Makhlouf et al. [53]	-	-	3.42 × 10^−6^	7.5 × 10^8^	8.3 × 10^6^
Unni and Seshaiyer [54]	-	-	1.0 × 10^−7^	4.8 × 10^2^	41.7
Gurcan et al. [55]	-	-	3.422 × 10^−9^	-	-
Khajanchi et al. [56]	-	-	2.2 × 10^−8^	1.3 × 10^4^	-
Pillis et al. [57]	-	-	1.0 × 10^−7^	1.30 × 10^4^	-
Kuznetsov et al. [58]	7.2	0.9997	0.00216	1.36 × 10^4^	0.0412
Pillis et al. [59]	-	-	1	0.33	0.2
Kuznetsov et al. [60]	1.387 × 10^−1^	0.9982	2.496 × 10^−4^	0.1950	0.5010
Sajid et al. [61]	10^−3^	-	-	-	-
Ottesen et al. [62]	10^−6^	-	-	-	-
Our default value	2.32 × 10^−4^	0.857	3 × 10^−5^	50.25	50
**Parameter**	$a_{y}$	$K_{y}$	$\beta_{y}$	$d_{y}$	$r_{y}p_{y}$
**Units**	**day^−1^**	**cell**	**cell^−1^ day^−1^**	**day^−1^**	**cell^−1^ day^−1^**
Almocera et al. [63]	1.62	10^9^	0.96	0.6	4.88 × 10^−8^
Ghosh, I. [64]	-	-	0.52	0.65	5.74 × 10^−4^
Hernandez-Vargas et al. [65]	8.57	-	1.26 × 10^5^	-	1.89 × 10^−6^
Our default value	1.15 × 10^−1^	3 × 10^3^	0.933	1 × 10^−2^	3.7 × 10^−4^
**Parameters**	$r_{y}$	$p_{y}$	$r_{y}\left(1-p_{y}\right)$	α	ε
**Units**	**cell^−1^ day^−1^**	**-**	**cell^−1^ day^−1^**	**cell day^−1^**	**day^−1^**
Almocera et al. [63]	-	-	-	2 × 10^5^	2 × 10^−1^
Ghosh, I. [64]	-	-	3 × 10^−7^	0.1	1
Hernandez-Vargas et al. [65]	-	-	10^−6^	-	-
Our default value	5.2 × 10^−4^	0.718	1.5 × 10^−4^	50.25	50
